# NPTX1在胸腺瘤患者中的表达及诊断价值

**DOI:** 10.3779/j.issn.1009-3419.2021.102.03

**Published:** 2021-01-20

**Authors:** 鑫 杜, 磊 于, 凌 杨, 丁方 曹, 颖 张

**Affiliations:** 1 100730 北京，首都医科大学附属北京同仁医院胸外科 Department of Thoracic Surgery, Beijing Tongren Hospital, Capital Medical University, Beijing 100730, China; 2 100730 北京，首都医科大学附属北京同仁医院中心实验室 Central Laboratory, Beijing Tongren Hospital, Capital Medical University, Beijing 100730, China; 3 100730 北京，首都医科大学附属北京同仁医院病理科 Department of Pathology, Beijing Tongren Hospital, Capital Medical University, Beijing 100730, China

**Keywords:** 胸腺瘤, 神经细胞正五聚体1, 微阵列分析, 受试者工作特征曲线, 肿瘤标志物, Thymoma, Neuronal pentraxin 1, Microarray analysis, Receiver operating characteristic curve, Tumor marker

## Abstract

**背景与目的:**

胸腺瘤是原发于前纵隔最常见的恶性肿瘤，但对于胸腺瘤的诊断没有特异性的实验室指标。本研究旨在通过mRNA微阵列分析技术筛选出对胸腺瘤诊断有提示作用的肿瘤标志物，并加以证实。

**方法:**

前期对31份胸腺瘤和瘤旁胸腺组织进行mRNA微阵列分析，发现胸腺瘤中神经细胞正五聚体1（neuronal pentraxin 1, *NPTX1*）基因转录水平上调超过4倍。为进一步验证上述结果，采用实时荧光定量聚合酶链反应（polymerase chain reaction, PCR）法、免疫组化法和酶联免疫吸附测定法（enzyme-linked immunosorbent assay, ELISA）对60例胸腺瘤患者和30例胸腺囊肿患者*NPTX1*基因的转录和表达水平进行检测，并应用受试者工作特征曲线（receiver operating characteristic curve, ROC）分析其在胸腺瘤中的诊断价值。

**结果:**

胸腺瘤组织中NPTX1 mRNA转录水平明显高于对照组胸腺组织[(2.88±1.02) *vs* (1.35±0.47), *P* < 0.001]；胸腺瘤组织中NPTX1蛋白的表达水平明显高于对照组胸腺组织（2 *vs* 1, *P* < 0.001）；术前胸腺瘤患者血清中NPTX1蛋白含量明显高于对照组胸腺囊肿患者[(1, 018.29±209.38) pg/mL *vs* (759.95±66.02) pg/mL, *P* < 0.001]；血清中NPTX1蛋白含量以842.22 pg/mL为诊断节点，诊断胸腺瘤的敏感性为85.00%，特异性为93.33%，ROC曲线下面积为0.902。

**结论:**

NPTX1在胸腺瘤患者中高表达，且对胸腺瘤有诊断价值。

## Abstract (en)

**Background and objective:**

Thymomas are the most common primary malignant tumors of anterior mediastinal. However, there are no specific laboratory indicator for the diagnosis the diagnosis of thymoma. The aim of this study was to screen out a tumor marker for diagnosis of thymoma by mRNA microarray analysis and confirmed it.

**Methods:**

By mRNA microarray analysis of 31 thymomas and peritumoral thymic tissues, we found that the transcription level of neuronal pentraxin 1 (*NPTX1*) gene was up-regulated more than 4 times in thymomas. To further verify the above results, we detected the transcription and expression level of NPTX1 in 60 thymoma and 30 thymic cyst patients by quantitative Real-Time polymerase chain reaction (PCR), immunohistochemistry and enzyme-linked immunosorbent assay (ELISA). Furthermore, the diagnostic value of NPTX1 in thymoma by receiver operating characteristic curve (ROC) was analyzed.

**Results:**

The transcription level of NPTX1 mRNA in thymoma tissues was significantly higher than that in the thymic tissues of control group [(2.88±1.02) *vs* (1.35±0.47), *P* < 0.001); The expression level of NPTX1 in thymoma tissues was significantly higher than that in the thymic tissues of control group (2 *vs* 1, *P* < 0.001); The preoperative serum level of NPTX1 protein in thymoma patients were significantly higher than that in the thymic cyst patients of control group [(1, 018.29±209.38) pg/mL *vs* (759.95±66.02) pg/mL, *P* < 0.001]; At the threshold of 842.22 pg/mL, sensitivity and specificity of NPTX1 as a serologic marker were 85.00% and 93.33%, respectively for thymoma. ROC showed that the area the under curve (AUC) of NPTX1 was 0.902.

**Conclusion:**

NPTX1 was highly expressed in thymoma patients, and had diagnostic value for thymoma.

胸腺瘤起源于胸腺上皮细胞，为原发于前纵隔中最常见肿瘤，年发病率为1.4/1, 000, 000^[1]^。因其各主要病理亚型均可表现出侵袭性，所以胸腺瘤均被定义为恶性肿瘤^[2]^。约1/3的胸腺瘤患者在诊断时没有任何症状，1/3的患者有局部压迫症状，1/3的患者合并副瘤综合征^[3]^。目前对于胸腺瘤的诊断没有特异性和针对性的辅助检查和检验指标，主要采用影像学检查来明确有无前纵隔占位的存在。但有时又难与发生于前纵隔的畸胎瘤、淋巴瘤、胸腺囊肿等相鉴别，还需行穿刺活检、胸腔镜、纵隔镜等有创检查来进一步明确病理诊断。

神经细胞正五聚体1（neuronal pentraxin 1, *NPTX1*）基因位于17q25上，其编码的NPTX1蛋白是一个4, 750 kDa的分泌糖蛋白^[4]^。NPTX1在中枢神经系统中高度表达，在神经细胞的突触可塑性和神经突触生长过程中发挥重要作用^[5-7]^。此外，研究还发现NPTX1参与细胞程序性凋亡^[8-10]^及癌症进展^[11-16]^。前期我们通过mRNA微阵列分析发现NPTX1 mRNA在胸腺瘤组织中的转录水平明显高于瘤旁组织^[17]^，但国际上暂无相关研究。本研究通过检测胸腺瘤组织中*NPTX1*基因的转录、蛋白表达情况和手术前后血清中NPTX1蛋白含量变化，并绘制受试者工作特征曲线（receiver operating characteristic curve, ROC），明确NPTX1在胸腺瘤患者中的表达情况及临床诊断价值。

## 1 材料与方法

### 1.1 mRNA微阵列分析及验证

用包含34, 000个人类mRNA探针的微阵列芯片（博奥生物股份有限公司，中国）对31份经手术切除的新鲜胸腺瘤和瘤旁胸腺组织进行分析，操作流程按照说明书进行如下：①使用TRIzol试剂（Invitrogen公司，美国）分别从新鲜肿瘤组织和瘤旁胸腺组织中提取总RNA，使用mirVana miRNA Isolation Kit（Ambion公司，美国）纯化；②使用CapitalBio cRNA扩增与标记试剂盒（博奥生物股份有限公司，中国）对RNA进行扩增和标记；③用Agilent杂交盒（Agilent公司，美国）配置杂交体系，并在杂交炉中45 oC杂交过夜；④使用GeneSpring软件V13.0（Agilent公司，美国）对数据进行归一化和差异分析，相对瘤旁组织，胸腺瘤组织中基因表达差异超过2倍，且*P* < 0.05的基因被认为是差异表达基因；⑤用实时荧光定量聚合酶链反应（polymerase chain reaction, PCR）法对差异表达的mRNA进行验证。取材均获得患者的知情同意，研究工作也通过了首都医科大学附属北京同仁医院伦理委员会的审查和批准。

### 1.2 研究对象及材料

收集2017年1月-2019年12月于首都医科大学附属北京同仁医院胸外科行手术治疗的60例胸腺瘤患者为实验组，30例不伴任何临床症状的胸腺囊肿患者作为对照组。总体年龄范围22岁-71岁，男女比例1:1。其中胸腺瘤组中A型胸腺瘤4例，AB型、B1型-B3型胸腺瘤分别为14例；合并重症肌无力的患者19例，合并其他自身免疫病的患者3例（2例合并皮肌炎、1例合并心肌炎）。纳入标准：①初诊；②术前未接受放化疗；③术后病理为胸腺瘤和胸腺囊肿；④围术期未出现严重的并发症；⑤所有患者的临床资料、病理分型分期及随访信息完整。

将一部分手术切除的新鲜胸腺瘤和胸腺组织在保证无菌的条件下储存在液氮中，待后续实验用。另一部分用10%中性福尔马林常温固定24 h，常规脱水、透明、浸蜡、石蜡包埋，制作成石蜡块，待后续实验用。

患者手术前和手术后1周的血清样本取自入院检查所需采血的残余血样。抽取患者空腹静脉血于美国BD分离胶促凝管中，4 oC冰箱中保存，2 h内运回实验室以3, 000 rpm离心15 min，收集血清并分装，储存于-80 oC冰箱中，以备后续实验使用。

### 1.3 研究方法

#### 1.3.1 实时荧光定量PCR法检测样本中NPTX1 mRNA的转录水平

取液氮冻存的新鲜组织样本50 mg于研钵中充分研磨，用TRIzol法提取总RNA。用Nano Drop 2000系统检测提取的各样本中RNA浓度，根据浓度结果算取含100 ng的RNA提取液作为mRNA模板，加入Oligo（dT），在逆转录酶的作用下反转录成cDNA。配制20 μL反应体系，使用ABI 7500 Real-Time PCR System平台扩增，反应条件为：95 oC预变性、2 min；95 oC、20 s，60 oC、40 s，共40个循环。循环结束后做溶解曲线并读取Ct值。20 μL反应体系（北京金诺信达生物科技有限公司，中国）如下所示：2×SG Green I qPCR Mix 10 μL，PCR Forward Primer（引物序列CAAACTTTGCAATCGCTCAA）（10 μmol/L）1 μL，PCR Reverse Primer（引物序列GATCCTTGAGGCTGTTGGTC）（10 μmol/L）1 μL，DNA模板1 μL，ddH_2_O 7 μL，共20 μL。

以β-actin为内参，取每个样品3个复孔Ct值的均值记为该样品的Ct值。各样本目的基因的Ct值与内参的Ct值的差，即为该样本的ΔCt。取对照组胸腺组织中的一个样本为标准品，各样本的ΔCt值减去标准品的ΔCt值的差，即为该样本的ΔΔCt。用2^-ΔΔCt^计算出各样本中目的基因的相对含量进行统计学分析。

#### 1.3.2 免疫组化法检测样本中NPTX1蛋白表达水平并评分

将样本组织蜡块连续切片、脱蜡、水合，3%过氧化氢消除内源性过氧化物酶活性。蒸馏水冲洗，PBS浸洗5 min，进行抗原修复。PBS洗涤后用血清封闭，室温孵育10 min，倾去血清，滴加1:50稀释的NPTX1鼠单克隆抗体（货号：sc-374199，Santa Cruz公司，美国），37 oC孵育90 min。PBS洗涤后加入过氧化物酶标记的山羊抗鼠IgG（货号：KIHC-5，Proteintech公司，美国），室温孵育30 min。再次PBS洗涤后进行显色反应，待适宜时间用蒸馏水水洗终止反应。苏木素（货号：H8070，索莱宝科技有限公司，中国）复染细胞核，流水冲洗蓝化。逐级脱水，二甲苯透明2次，中性树胶封固后，显微镜下阅片。

由两位对患者信息不知情的病理医生对染好的石蜡切片中NPTX1的表达进行评分，染色强度为阴性记0分、可疑阳性记1分、弱阳性记2分、中阳性记3分、强阳性记4分。每位病理医生对每个标本取4个视野进行评分，并给出平均得分。最终得分为两位病理医生评分的平均分。

#### 1.3.3 酶联免疫吸附法（enzyme linked immunosorbent assay, ELISA）检测血清中NPTX1蛋白含量

将试剂恢复到室温，使用前充分混匀。在酶标板上分别设标准孔、待测样品孔和空白孔，且均设置复孔。标准孔中加血清50 μL。待测样品孔中用40 μL的样品稀释液（北京金诺信达生物科技有限公司，中国）将10 μL血清样品稀释5倍。空白孔中加样品稀释液50 μL。除空白孔外，每孔均需加入100 μL酶标试剂（北京金诺信达生物科技有限公司，中国）。酶标板封膜后置恒温箱中37 oC孵育90 min。去除封板膜，弃液并甩干。板孔中加入稀释后的洗涤液，静置30 s后弃去，重复上述洗板5次后，在吸水纸上将液体拍干。板孔中分别加入显色剂A、B（北京金诺信达生物科技有限公司，中国）各50 μL，震荡混匀后，于37 oC恒温箱中避光静置15 min。在板孔中加入50 μL终止液后反应终止，蓝色变为黄色。在终止显色15 min以内，以空白孔调零，450 nm波长测量各孔的吸光度。

### 1.4 统计学分析

数据分析采用SPSS 26.0统计软件，实时荧光定量PCR结果采用独立样本*t*检验进行分析，结果以均数±标准差（Mean±SD）表示；免疫组化评分采用*Mann-Whitney U*检验进行分析，结果以中位数表示；ELISA检测结果分别采用*t*检验和配对*t*检验进行分析，结果以均数±标准差（Mean±SD）表示；*P* < 0.05表示差异有统计学意义；绘制ROC，并计算曲线下面积、敏感性和特异性，评价血清中NPTX1蛋白作为胸腺瘤肿瘤标志物的诊断效能。

## 2 结果

### 2.1 mRNA微阵列分析结果及验证

mRNA微阵列分析结果显示，与瘤旁胸腺组织相比，胸腺瘤组织中共有825个基因出现转录水平异常，且差异超过2倍。其中有229个基因转录水平上调，596个基因转录水平下调。*NPTX1*是其中的一个上调基因，且上调水平超过4倍。通过PCR验证证实，在胸腺瘤组织中NPTX1 mRNA的转录水平较瘤旁胸腺组织明显升高[(3.14±1.26) *vs* (2.08±0.72)]，差异有明显统计学意义（*P* < 0.001）。见图 1。

**图 1:**
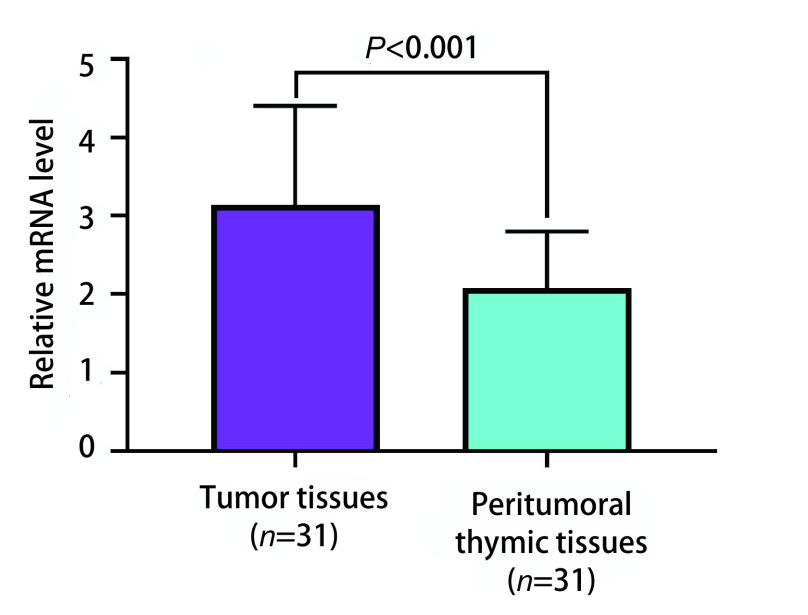
PCR证实胸腺瘤组织中NPTX1 mRNA的转录水平明显高于瘤旁胸腺组织 Confirmed by PCR, the transcription level of NPTX1 mRNA in thymoma tissues was significantly higher than that in peritumoral thymic tissues. PCR: polymerase chain reaction.

### 2.2 NPTX1 mRNA在组织中的表达分析

实时荧光定量PCR结果表明，胸腺瘤组织中NPTX1 mRNA的转录水平高于对照组胸腺组织[(2.88±1.02) *vs* (1.35±0.47)]，差异有明显统计学意义（*P* < 0.001）。见图 2。

**图 2:**
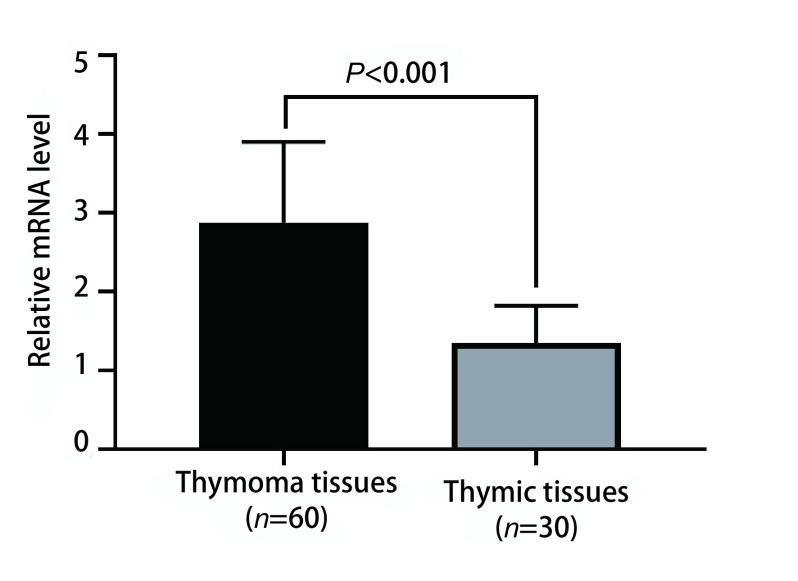
胸腺瘤组织中NPTX1 mRNA转录水平明显高于对照组胸腺组织 The transcription level of NPTX1 mRNA in thymoma tissues was significantly higher than that in thymic tissues of control group

### 2.3 NPTX1蛋白在组织中的表达分析

对胸腺瘤组织和对照组胸腺组织的染色评分进行统计学分析，发现胸腺瘤组织中NPTX1蛋白的免疫组化评分高于胸腺组织（2 *vs* 1），差异有明显统计学意义（*P* < 0.001）。见图 3。

**图 3:**
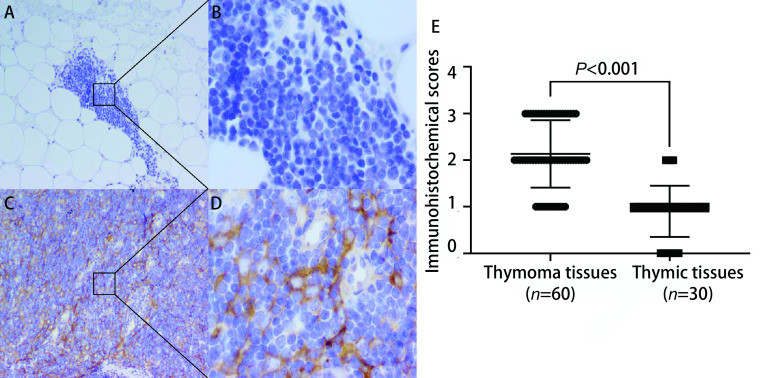
免疫组化染色结果及评分。A、B：NPTX1表达结果为阴性的1例胸腺组织（A: ×100; B: ×400）；C、D：NPTX1表达结果为阳性的1例胸腺瘤组织（C: ×100; D: ×400）；E：NPTX1在胸腺瘤组织中的免疫组化评分显著高于胸腺组织。 The results and scores of immuno-histochemistry. A, B: A thymus tissue in which NPTX1 expression was negative (A: ×100; B: ×400); C, D: A thymoma tissue in which NPTX1 expression was positive (C: ×100; D: ×400); E: The immunohistochemical score of NPTX1 in thymoma tissues was significantly higher than that in thymic tissues.

### 2.4 NPTX1蛋白在血清中的含量及诊断效能分析

ELISA检测血清中NPTX1蛋白含量显示，胸腺瘤组患者术前血清中NPTX1蛋白含量高于对照组胸腺囊肿患者术前[(1, 018.29±209.38) pg/mL *vs* (759.95±66.02) pg/mL]，差异有明显统计学意义（*P* < 0.001）（图 4A）。胸腺瘤组患者术前血清中NPTX1蛋白含量高于术后[(1, 018.29±209.38) pg/mL *vs* (856.67±165.36) pg/mL]，差异有明显统计学意义（*P* < 0.001）（图 4B）。但对照组胸腺囊肿患者术前与术后血清中NPTX1蛋白含量无明显差异[(759.95±66.02) pg/mL *vs* (752.63±86.83) pg/mL]（*P*=0.54）（图 4C）。以血清中NPTX1蛋白含量作为诊断胸腺瘤的指标绘制ROC（图 5），曲线下面积为0.902（95%CI: 0.837-0.968）。以842.22 pg/mL为诊断节点，敏感性为85.00%，特异性为93.33%。准确性为87.78%，阳性预测值为96.23%，阴性预测值为75.67%。

**图 4:**
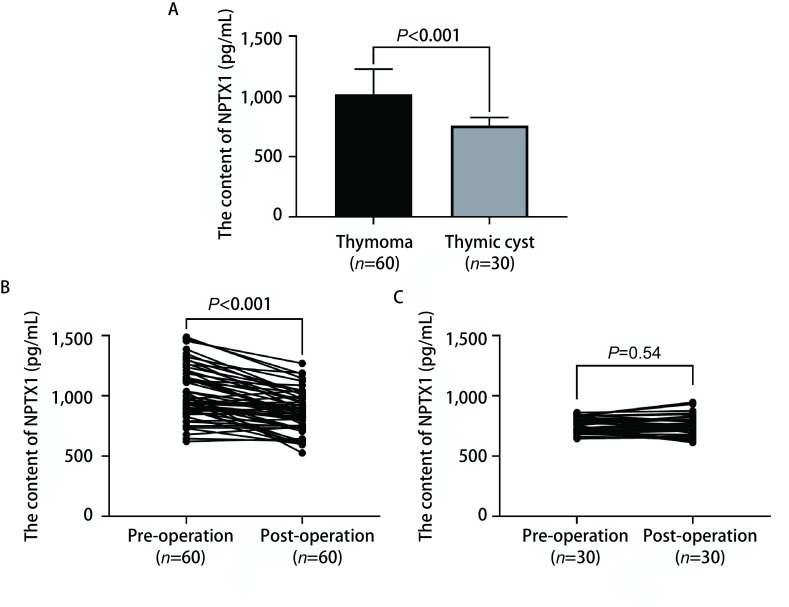
血清中NPTX1蛋白含量比较。A：术前胸腺瘤患者血清中NPTX1蛋白含量明显高于对照组胸腺囊肿患者；B：胸腺瘤患者术后血清中NPTX1蛋白含量较术前明显降低；C：对照组胸腺囊肿患者术后血清中NPTX1蛋白含量较术前未见明显差异。 Comparison of NPTX1 protein content in serum. A: Before operation, serum levels of NPTX1 protein in patients with thymoma were significantly higher than those in patients with thymoma cyst in the control group; B: The content of NPTX1 in serum of thymoma patients was significantly decreased after operation; C: There was no significant difference about NPTX1 content in serum of thymic cysts patients before and after operation.

**图 5:**
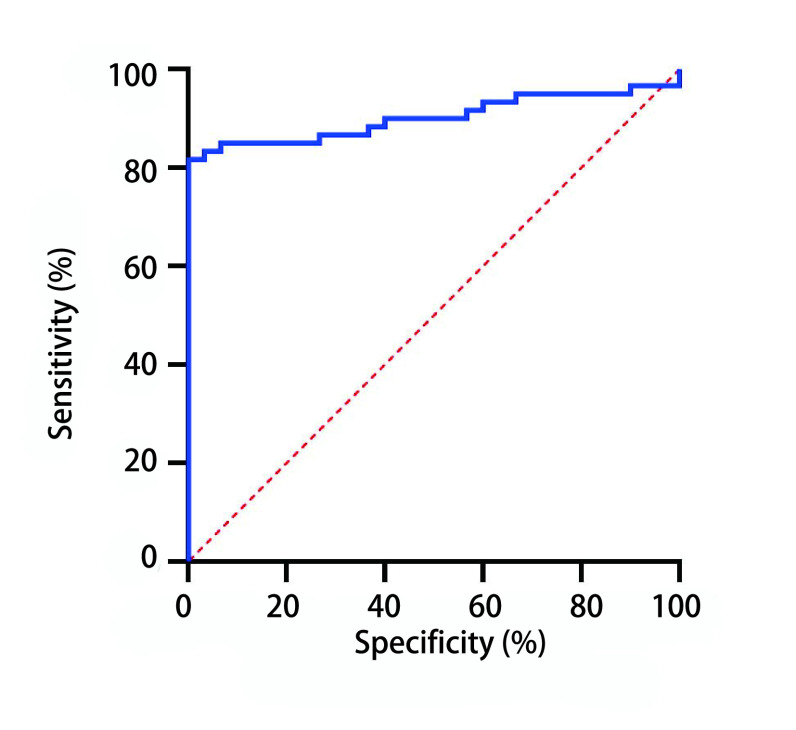
血清中NPTX1蛋白含量诊断胸腺瘤的ROC曲线 The ROC curve of serum NPTX1 protein in diagnosis of thymoma. ROC: receiver operating characteristic curve.

## 3 讨论

胸腺瘤作为一种恶性肿瘤，早期发现、早期诊断、早期治疗对于胸腺瘤预后是极其有利的，但因胸腺瘤生长缓慢、起病隐匿，绝大部分患者又无显著的特征性临床症状以及缺乏针对性检查手段，所以很容易被误诊或漏诊，导致病情的延误。因此对于开发胸腺瘤的肿瘤标志物临床意义重大。

前期我们利用mRNA微阵列分析技术发现胸腺瘤组织中*NPTX1*基因转录水平上调。本研究通过扩大样本量进行对照分析，利用荧光定量PCR法、免疫组化法和ELISA法证实了在胸腺瘤患者的瘤体组织及血清样本中*NPTX1*基因的转录和表达水平明显高于对照组。同时研究发现术前胸腺瘤患者血清中NPTX1蛋白含量明显高于对照组胸腺囊肿患者，且术后显著下降，而手术前后对照组胸腺囊肿患者血清中NPTX1蛋白含量未见明显变化。结合ROC分析结果，考虑NPTX1在胸腺瘤患者中高表达，且血清中NPTX1蛋白可作为诊断胸腺瘤的肿瘤标志物，诊断节点为842.22 pg/mL。

有研究^[11]^发现，circRNA（hsa_circ_0070269）通过使肝癌细胞中miR-182海绵化而增加了NPTX1的表达，抑制肝癌细胞的增殖和侵袭。此外Peng等^[15]^发现，NPTX1过表达抑制结肠癌细胞中的cyclin A2和CDK2的表达，从而调控Rb-E2F信号通路，起到抑制结肠癌细胞生长的作用。因此我们猜想，胸腺瘤生长相对缓慢的临床特征也可能与胸腺瘤细胞中NPTX1呈高表达相关。

综上所述，本研究发现NPTX1在胸腺瘤患者中高表达，且血清中NPTX1作为胸腺瘤的诊断指标有较高的敏感性和特异性，表现出了良好的临床应用前景，使之有望成为诊断胸腺瘤的肿瘤标志物。但对于其在胸腺瘤患者中的作用机制，还需要通过细胞系和动物模型实验来进一步阐明。
